# Rush Medical College

**Published:** 1845-02

**Authors:** J. H. Bird


### ILLINOIS

### MEDICAL & SURGICAL JOURNAL.

VOL. I. FEBRUARY, 1845. NO. 11.

### RUSH MEDICAL COLLEGE.

### PROF. BRAINARD'S SURGICAL CLINIC. JAN. 2d, 1845.

[Reported for the Journal, by J. H. Bird.]

Case 1.—Mr. W. I———, having ectropion, occasioned by a burn, presented himself for relief. Dr. Brainard remarked that within the last few years, numerous operations have been performed, for the restoration of different parts of the face, destroyed by burns, and other causes. These were formerly called Taliacotian operations, from Taliacotius, an Italian surgeon, who attained great celebrity in the restoration of lost noses; but as he is not the inventor of the operation, and as plastic operation is more appropriate, it is now called by that name, and plastic surgery is recognized as a distinct branch of the art. If the operation is performed upon the nose, it is called rhinoplastic; if upon the eye, blepharoplastic, &c., according to the part. In the present case, the deformity was occasioned by the patient falling upon a bed of coals, producing a burn of great extent, causing a destruction of the soft parts to a considerable depth. In future lectures, I will speak of the different effects resulting from burns. There is, in the present case, a cicatrix, extending from the eye downwards upon the face, producing complete eversion of the lower lid. In extensive burns, there is sloughing of the part; and extensive ulcers are formed, which granulate. These granulations contract, and the cicatrix is formed. There is a great tendency on the part of the cicatrix to contract; in case of a burn upon the hand, the fingers will be strongly flexed, or applied upon the dorsum of the hand; if upon the fore-arm, it will be flexed upon the arm; if upon the neck, the head will be drawn towards the breast, &c. The knowledge of this contractibility is very important in the dressings of burns. In the present case, you observe the effect of the contraction upon the eyelid: it is not destroyed, but the inferior edge is planted against the globe of the eye, and the free edge is adherent at the summit of the cheek. It has been proposed to divide the cicatrix and sound skin adjacent, but it has met with poor success.

The idea of the plastic operation originated in India, where the punishment inflicted upon criminals, as cutting off of noses and ears, gave so frequent opportunities for their remedy. The part to be applied can be taken from the adjacent parts, or from distant ones, as from the arm. This latter is the Taliacotian method, but at the present day is rarely repeated. Wonderful stories have been told of noses taken from other individuals adhering, but to these we give no credence. Another method is by inoculation, that is, by removing distant parts to the face; as from the arm to the neck, and when adhesion will allow, again removing it to the face. This may do, in cases where the face and adjacent parts are too much injured to allow of their use. No particular rule can be laid down, but, as occasion may call, the surgeon will have to adopt the method that may seem best.

I would observe, that instances are on record, of parts entirely divided, uniting. I had a case, where the pulp of the finger had been entirely cut off, the piece washed free from dirt, and re-applied. On application to me, I for curiosity took the piece off, and re-applied it; but I found, although there seemed to be a union, yet the part had wasted almost entirely away.

There are many ways of performing the operation for the relief of ectropion. I have seen Velpeau use a method, which consists in removing a portion of the integuments at the inner or nasal side, then drawing the edges of the wound together, so as to fill the vacant place; a slight improvement was noticed, but the result was not satisfactory. Another method is, to excise a portion of the conjunctiva; another still, to cut out portion a of the lid, in the shape of the letter V.

These may be admissible in slighter cases, but in more perfect eversion, as in the present case, it becomes necessary to supply the place of the destroyed part. The piece to be used can be taken from the nose, face, forehead or temporal region. In this case, owing to the extent of the burn, there remains to us but the two last. There has been another operation proposed, which consists in excising a portion of the integuments, at the external angle uniting the edges of the wound, thus drawing the lids outwards, and elevating the everted one. We intend to combine the two modes. By removing a piece from the temple, the contraction of the cicatrix will have a tendency to restore the palpebra to its position; while the inserting the piece, thus removed, beneath it, will perfectly effect this object. The operation combining the two objects, has not, as far as we are aware, been performed or proposed by any surgeon.

We will commence the operation by dissecting up the eyelid, making one incision at the inferior margin of the tarsal cartilage, it will then resume its true position. Then dissect up a portion of skin without the external angle, and turning the flap upon its base about one fourth of a circle, we will place in in the incision already made under the eyelid, keeping the parts in contact by means of interrupted sutures, three above and two below. Owing to the contractility of living tissue, we will make our flap about one third larger than the chasm it is to fill. The tissue of a cicatrix possesses but little vitality; if blisters are applied to its surface, the vesicated parts are very long in healing. So the sound portion of skin will be much longer in uniting to the cicatrized portion.

Fergusson, and other authors say, that before the new portion shall be brought in contact with the other, hæmorrhage must have partially ceased, and the wound be cleansed. I do not think it necessary to wait for the cessation of the hæmorrhage, but will merely cleanse the wound from any coagula that may have formed, and apply the flap. Adhesive straps and pressure, should not be employed in the dressings, as they tend to interrupt the limited circulation in the parts. The pedicle of the flap should be broad enough to admit of sufficient circulation to preserve its vitality. And this depends upon its situation; in the present instance and in plastic operations upon the nose, the circulation, owing to the number of arteries, is quite active.

The after treatment and dressings are quite simple; merely defensive. No cold applications should be used, as they tend to check the circulation.

The Dr. then performed the operation. The new portion having been nicely adapted to the incision, the proper dressings were then applied, and the patient dismissed. He then remarked that the wound would heal generally by the first intention, and that the fullness at the external angle, from the torsion of the flap, might be treated by excision, if necessary. As the patient had a deformity of the mouth produced by the same cause, being a contraction of the opening of the lips, he proposed operating in a few days upon it, by increasing the size of the opening. He continued his remarks. The mouth could be contracted by various causes, as psoriasis labialis, &c.; mechanical means, as various plugs, would not prevent the contraction; an operation was necessary. The operation recommended by Dieffenbach, has been quite successful. It consists in excising the integuments down to the mucous membrane, drawing this over the edges of the wound, and by stiches, producing an adherence of it to the skin. The contraction in the present case, is to one-third of the opening, and is external; the mucous membrane being intact. As the cicatrix is not in as favorable a state as in the other case, I am not so certain as to the result. At a subsequent period the operation was performed.

Jan. 20. The patient presented himself. The wound on the temple had nearly healed, and the flap had entirely adhered in its position. The operation had been quite successful. The wound at the corner of the mouth had nearly healed. All things being so favorable, the patient was permitted to leave for his home in the country.

---

Case 2.—Mr. ——, having a tumor upon the breast, presented himself for its removal. Dr. Brainard remarked that it was a pediculated vascular tumor, of very easy removal. The Dr., by two incisions quite expeditiously removed it; the edges of the wound were united, by stiches and adhesive straps; the proper dressings applied, and patient dismissed.

On examination, the tumor presented a fungoid vascular appearance, having the cancerous odor, without the peculiar sound in cutting it. The Dr. considered it of the variety of tumors, called by Warren, keloides.

---

Case 3.—Mr. ——, afflicted with chronic catarrhal opthalmia. Dr. B. remarked, that this was another case of that common disorder, chronic conjunctivitis with blepharitis. We will prescribe the blue mass, 3 grs., to be taken each night, as a gentle laxative; and the ointment of sulphate zinc, and simple cerate, in the proportion hitherto mentioned, to be applied in the usual manner.

